# Gene Expression Signature Analysis Identifies Vorinostat as a Candidate Therapy for Gastric Cancer

**DOI:** 10.1371/journal.pone.0024662

**Published:** 2011-09-09

**Authors:** Sofie Claerhout, Jae Yun Lim, Woonyoung Choi, Yun-Yong Park, KyoungHyun Kim, Sang-Bae Kim, Ju-Seog Lee, Gordon B. Mills, Jae Yong Cho

**Affiliations:** 1 Division of Cancer Medicine, Department of Systems Biology, The University of Texas MD Anderson Cancer Center, Houston, Texas, United States of America; 2 Department of Medical Oncology, Yonsei University College of Medicine, Gangnam Severance Hospital, Seoul, Korea; 3 Department of Cancer Biology, The University of Texas MD Anderson Cancer Center, Houston, Texas, United States of America; 4 Department of Veterinary Physiology and Pharmacology, Texas A&M University, College Station, Texas, United States of America; IIT Research Institute, United States of America

## Abstract

**Background:**

Gastric cancer continues to be one of the deadliest cancers in the world and therefore identification of new drugs targeting this type of cancer is thus of significant importance. The purpose of this study was to identify and validate a therapeutic agent which might improve the outcomes for gastric cancer patients in the future.

**Methodology/Principal Findings:**

Using microarray technology, we generated a gene expression profile of human gastric cancer–specific genes from human gastric cancer tissue samples. We used this profile in the Broad Institute's Connectivity Map analysis to identify candidate therapeutic compounds for gastric cancer. We found the histone deacetylase inhibitor vorinostat as the lead compound and thus a potential therapeutic drug for gastric cancer. Vorinostat induced both apoptosis and autophagy in gastric cancer cell lines. Pharmacological and genetic inhibition of autophagy however, increased the therapeutic efficacy of vorinostat, indicating that a combination of vorinostat with autophagy inhibitors may therapeutically be more beneficial. Moreover, gene expression analysis of gastric cancer identified a collection of genes (*ITGB5, TYMS, MYB, APOC1, CBX5, PLA2G2A,* and *KIF20A*) whose expression was elevated in gastric tumor tissue and downregulated more than 2-fold by vorinostat treatment in gastric cancer cell lines. In contrast, *SCGB2A1, TCN1, CFD, APLP1,* and *NQO1* manifested a reversed pattern.

**Conclusions/Significance:**

We showed that analysis of gene expression signature may represent an emerging approach to discover therapeutic agents for gastric cancer, such as vorinostat. The observation of altered gene expression after vorinostat treatment may provide the clue to identify the molecular mechanism of vorinostat and those patients likely to benefit from vorinostat treatment.

## Introduction

Gastric cancer is the fourth most common cancer and the second leading cause of cancer death in the world [1], with an overall survival of about 10 months [2]–[4]. Treatment for gastric cancer may include chemotherapy, surgery and radiation therapy. Unfortunately, current chemotherapy-based treatments for advanced gastric cancer demonstrate disappointing results [2]–[4]. Indeed, complete remissions are rare or only last very shortly.

Several targeted agents that confer survival advantages in other cancer types have been under investigation in gastric cancer. While some early clinical studies using vascular endothelial growth factor receptor (VEGFR) and epithelial growth factor receptor (EGFR) -1 inhibitors, such as cetuximab and bevacizumab, have shown somewhat activity, there is rarely an actual survival benefit for the patients [5], [6]. One of the reasons may be that these studies did not select patients according to the presence of biomarkers. Recently, the Trastuzumab for Gastric Cancer (ToGA) trial noted that the addition of trastuzumab to chemotherapy led to a statistically significant improvement in progression-free survival (PFS) and overall survival (OS) of the approximately 20% of patients with disseminated gastric and gastroesophageal (GE) junction tumors overexpressing HER2 [7]. This emphasizes the need for targeted biological therapy and the search for biomarkers to select patients for clinical trials which may benefit survival. Despite some evidence of potential targets, including HER2 [8], [9], the efficacy of these biologically targeted therapies is not known and there is a lack of a standard targeted therapy for gastric cancer. Owing to the biological heterogeneity of gastric cancers, it is unlikely that there is a single ‘magic bullet’ cure. Molecular markers will be thus important in the future to predict patients' outcomes and tailoring treatments according to individual biology.

In the search for biomarkers, gene expression signature analysis has been used in diverse applications, such as for elucidating the mechanisms of biological pathways [10], classifying subtypes of a disease [11], predicting cancer prognosis [12] and profiling gene expression in response to specific drugs [13], [14]. Gene expression signature analysis can be done by using The Broad Institute's Connectivity Map (http://www.broadinstitute.org/cmap). The Connectivity Map aims to generate a map that links gene expression patterns associated with disease to corresponding patterns produced by drug candidates and genetic manipulations [15], [16]. This systems approach allows compounds to be screened against genome-wide disease signatures, rather than a preselected set of target genes. Drugs are paired with diseases using sophisticated pattern-matching methods with a high level of resolution and specificity. Although it leaves many open questions, the Connectivity Map has shown that genomic signature analysis can be used to recognize drugs with common mechanisms of actions, discover unknown mechanisms of action and identify potential new therapeutics [15], [16].

The purpose of this study was to identify potential new therapeutics for the treatment of gastric cancer. To do this, we first analyzed the genomic signature of human gastric cancer. The resultant gastric cancer gene signature was then used *in silico* by employing Connectivity Map analysis to identify therapeutic agents that could potentially be effective against this type of cancer. We further validated the top targeting drug for its efficacy in gastric cancer cell lines. We found that vorinostat, as a potential new drug, induced both apoptosis and autophagy in gastric cancer cells. Together, this study demonstrates that the Connectivity Map analysis can be used for the identification of therapeutic agents that may be successful in the treatment of a subset of gastric cancers.

## Methods

### Analysis of microarray data

For the Connectivity Map analysis, we used the microarray data of 65 gastric cancer patients, including 65 cancers and 19 normal gastric tissues, which were obtained from our previous work, Yonsei data [17]. Tumor specimens were collected from gastric cancer patients undergoing gastrectomy as a primary treatment. Tissue samples were examined by pathologists at the time of collection and stored in −80°C at the tissue bank until the start of the experiment. Total RNA was extracted from the fresh-frozen tissues by using a mirVana RNA isolation labeling kit (Ambion, Inc.). Primary microarray data is available in NCBI's Gene Expression Omnibus public database (microarray platform, GPL6884; microarray data, GSE 13861). Another gene expression profile was obtained from 69 gastric tissue samples, including 38 cancer and 31 non cancer stroma, of the Stanford Microarray Database (http://smd.stanford.edu, GSE13911), Stanford data. Gastric cancer-specific genes were selected by BRB-ArrayTools version 3.6.1 (Biometric Research Branch, National Cancer Institute, Bethesda, MD). Class comparison using two sample t-test (significance <0.001, 10,000 random permutation) identified gastric cancer specific genes and genes whose mean expression intensities were altered by at least two-fold compared to mean normal tissue gene expression were selected.

### Connectivity Map analysis

To identify potential drugs targeting gastric cancer, the gene lists of top 500 up regulated and the top 500 down regulated genes from the gastric cancer-specific genes were used (Table S1). The Connectivity Map analysis was conducted through the Web interface (http://www.broadinstitute.org/cmap) using version, build 02, which contains more than 7,000 expression profiles representing effects of 1,309 compounds on several cultured human cells [15], [16]. The Connectivity map shows functional connections between drugs, genes and disease. Drugs that produce disease-mimicking gene signatures can help identify pathways that represent potential therapeutic targets for that disease. Conversely, drugs that induce a ‘reverse’ signature, i.e. changes in gene expression in a direction opposite to that observed in the disease state, could represent new therapeutic agents. Candidate agents against a specific disease can be recognized by applying disease specific gene expression profile to the Connectivity Map analysis. We selected candidate drugs for validation *in vitro* on the basis of the connectivity score, correlation, and P-value.

### Chemicals and cell culture

Vorinostat was obtained from Merck and prepared as a stock solution in dimethylsulfoxide (DMSO). Chloroquine and bafilomycin A1 (Sigma) were dissolved in respectively water and DMSO. Human gastric cancer cell lines AGS, NCI-N87, and KATO-III were obtained from the American Type Culture Collection and were maintained according to their recommendations. Cells were cultured in RPMI 1640 supplemented with 10% fetal bovine serum, 100 U penicillin, and 100 µg/ml streptomycin at 37°C in 5% CO_2_.

### Cell growth, viability and cell cycle assays

For the 3-(4,5-dimethylthiazol-2-yl)-2,5-diphenyl tetrazolium bromide (MTT) assay, MTT was dissolved in PBS at 5 mg/ml. About 5×10^3^ cells were seeded in 96-well plates and allowed to attach overnight. The culture medium was then replaced with fresh medium containing the indicated concentrations of vorinostat or DMSO. After 72 h, 20 µl of MTT solution was added, and the plates were incubated at 37°C for 4 h. After incubation, 100 µl of DMSO was added to dissolve the formazan, and absorbance was read at 570 nm using a spectrophotometric microplate reader (Vmax kinetic microplate reader, Molecular Devices). The experiments were done in triplicate.

For crystal violet staining, cells were treated for 12 h, the medium was removed, and the cells were washed with PBS and then incubated for 30 min with 0.5% crystal violet (in 20% methanol and 80% double-distilled water). The cells were then washed three times with PBS. The remaining crystal violet was extracted in acetic acid for 5 min, and absorbance was measured at 595 nm using a spectrophotometric microplate reader.

To determine cell viability, cells were incubated with vorinostat for 72 h. Adherent cells were then detached from culture plates by trypsinization and combined with floating cells, centrifuged and suspended in 500 µl of propidium iodide (PI)-exclusive solution (not cell membrane penetrating) for 15 min at 4°C. Stained cells were monitored by flow cytometry (Beckman Coulter Cytomics FC 500).

For cell cycle analysis, cells were incubated with vorinostat for 24 h, collected and suspended in 500 µl of hypotonic solution (0.1% sodium citrate, 0.1% Triton X-100, 100 µg/ml RNase and 50 µg/ml PI) for 15 min at 4°C. PI-stained cells were monitored by flow cytometry. Cell cycle was analyzed by MultiCycle AV software.

### RNA isolation and microarray experiments

RNA isolation and microarray experiments were performed according to the protocol as previously described [17]. Total RNA was extracted from gastric cancer cell lines with or without vorinostat treatment using a mirVana RNA isolation kit (Ambion, TX, USA). The integrity of the large RNA fraction was determined with an Experion Bioanalyzer (Bio-Rad, CA, USA) as a surrogate for mRNA quality control. Total RNA was labeled and hybridized with human HT12 v.3 expression BeadChips according to the manufacturer's protocols (Illumina, CA, USA). After the BeadChips were scanned with an Illumina BeadArray Reader, the microarray data were normalized using the quantile normalization method in the Linear Models for Microarray Data package in the R language environment [18]. The expression level of each gene was transformed into a log_2_ base before further analysis. Cluster analysis was done with Cluster and Treeview [19].

### Western blot analysis

Cells were scraped in medium and spun down, and proteins were isolated using lysis buffer (50 mM HEPES, 150 mM NaCl, 1 mM EGTA and 10 mM sodium pyrophosphate (pH 7.4)) containing 100 mM NaF, 10% glycerol, 1.5 mM MgCl_2_, 1% Triton X-100 and protease inhibitor (Roche). Extracts were incubated on ice for 20 min and spun down at 20800 g for 20 min. Protein concentration was determined using BCA protein assay reagent (Pierce). Equal amounts of protein from each sample were separated by electrophoresis through SDS-PAGE and transferred to Hybond-C Super membrane (Amersham Pharmacia Biotech). Membranes were blocked for 1 h at room temperature in Tris-buffered saline containing 0.1% Tween-20 and 5% nonfat dry milk. Membranes were incubated overnight at 4°C with primary antibody diluted in 5% nonfat dry milk or 5% BSA in 1× Tris-buffered saline plus 0.1% Tween-20. Antibodies to LC3 (Novus Biologicals), active caspase-3 (Epitomics) and p62 (BD Biosciences) were used. Antibodies to α-tubulin and beclin-1 were from Cell Signaling Technology; antibody to β-actin was from Sigma. Membranes were then washed and incubated for 1 h at room temperature with peroxidase-conjugated secondary antibody (Cell Signaling Technology). Protein bands were visualized using enhanced chemiluminescence as described by the manufacturer (GE Healthcare).

### siRNA transfection

The siRNA target sequence for beclin-1, nontargeting siRNA (Risc Free) and Dharmafect 1 were purchased from Dharmacon. Cells were seeded in 10-cm dishes and transfected with siRNA 24 h later according to the manufacturer's protocol. The next day, cells were trypsinized and seeded in 6-cm or 96-well plates to obtain the same transfection efficiency. Protein expression levels were determined by western blot analysis.

### Transmission electron microscopy

Samples were fixed with a solution containing 3% gluteraldehyde plus 2% paraformaldehyde in 0.1 M cacodylate buffer, pH 7.3, for 1 hour. After fixation, the samples were washed and treated with 0.1% Millipore-filtered cacodylate buffered tannic acid, postfixed with 1% buffered osmium tetroxide for 30 min, and stained en bloc with 1% Millipore-filtered uranyl acetate. The samples were dehydrated in increasing concentrations of ethanol, infiltrated, and embedded in LX-112 medium. The samples were polymerized in a 70°C oven for 2 days. Ultrathin sections were cut in a Leica Ultracut microtome (Leica, Deerfield, IL), stained with uranyl acetate and lead citrate in a Leica EM stainer, and examined in a JEM 1010 transmission electron microscope (JEOL, USA, Inc., Peabody, MA) at an accelerating voltage of 80 kV. Digital images were obtained using AMT Imaging System (Advanced Microscopy Techniques Corp, Danvers, MA).

## Results

### Gene expression signature of gastric cancer

Table 1 represents the characteristics of the patients from Yonsei data [17]. Gastric cancers were mostly located in distal stomach and stage III/IV. Using the gene expression microarray data of those patients, we found 3,360 tumor-specific genes whose mean expression intensities were altered by at least two-fold compared to mean normal tissue gene expression (*P*<0.001, Figure S1). This set of 3,360 genes (i.e. gastric cancer-specific signature) was used for further *in silico* screening for potential therapeutic drugs for gastric cancer.

**Table 1:** Baseline characteristics of the gastric cancer patients (Yonsei data).

Characteristics	N = 65
**Age (yr)**	
Median (range)	63 (32–83)
**Sex (%)**	
Male: Female	46 (71): 19 (29)
**Subsite of tumor (%)**	
Cardia	5 (8)
Body	24 (37)
Antrum	32 (49)
Diffuse	4 (6)
**Histologic type of tumor (%)**	
Intestinal	23 (35)
Diffuse	32 (49)
Mixed	10 (16)
**Cancer stage, TNM class (%)**	
I	12 (18)
II	11 (17)
III	26 (40)
IV	16 (25)
**Adjuvant chemotherapy (%)**	
No: Yes	16 (25): 49 (75)

### Connectivity Map analysis identifies potential drugs targeting gastric cancer

To identify potential drugs targeting gastric cancer, the gastric cancer specific signature was used as input query into Connectivity Map as described in the ‘Method’ section. We specifically looked for compounds that had a signature inversely correlated with the gastric cancer-specific signature and identified multiple drugs which are summarized in Table 2. The ranking of candidate agents was established based on inverse correlation value and p-value. Table 2 (columns 2–4) shows the highest ranked compounds from Yonsei data. Connectivity Map analysis revealed that histone deacetylase (HDAC) inhibitors, including vorinostat and trichostatin A represent potential candidates for targeting gastric cancer. The phosphatidylinositol-3-kinase inhibitor LY294002, the phenothiazine trifluoperazine and the heat shock protein inhibitor tanespimycin were also identified as candidate target agents for gastric cancer. Next, we validated our findings by using an independent set of gene expression profile data from Stanford Microarray Database (Table 2; Stanford Data; columns 5–7). Connectivity Map analysis of this data set confirmed vorinostat as the top ranked candidate. In conclusion, Connectivity Map analysis identified vorinostat as a potential therapeutic agent for gastric cancer.

**Table 2:** Top 5 candidate drugs of gastric cancer from Connectivity Map analysis.

Rank	Yonsei Data (GSE31861)	Mean correlation (*r*)	p-value	Stanford Data (GSE13911)	Mean correlation (*r*)	p-value
1	vorinostat	−0.716	<0.00001	vorinostat	−0.714	<0.00001
2	trichostatin A	−0.643	<0.00001	trichostatin A	−0.653	<0.00001
3	tanespimycin	−0.539	<0.00001	tanespimycin	−0.527	<0.00001
4	trifluoperazine	−0.538	<0.00001	LY-294002	−0.52	0.00002
5	LY-294002	−0.45	<0.00001	0297417-0002B	−0.815	0.00052

### Vorinostat shows therapeutic efficacy *in vitro* in gastric cancer cell lines

**Figure 1:**
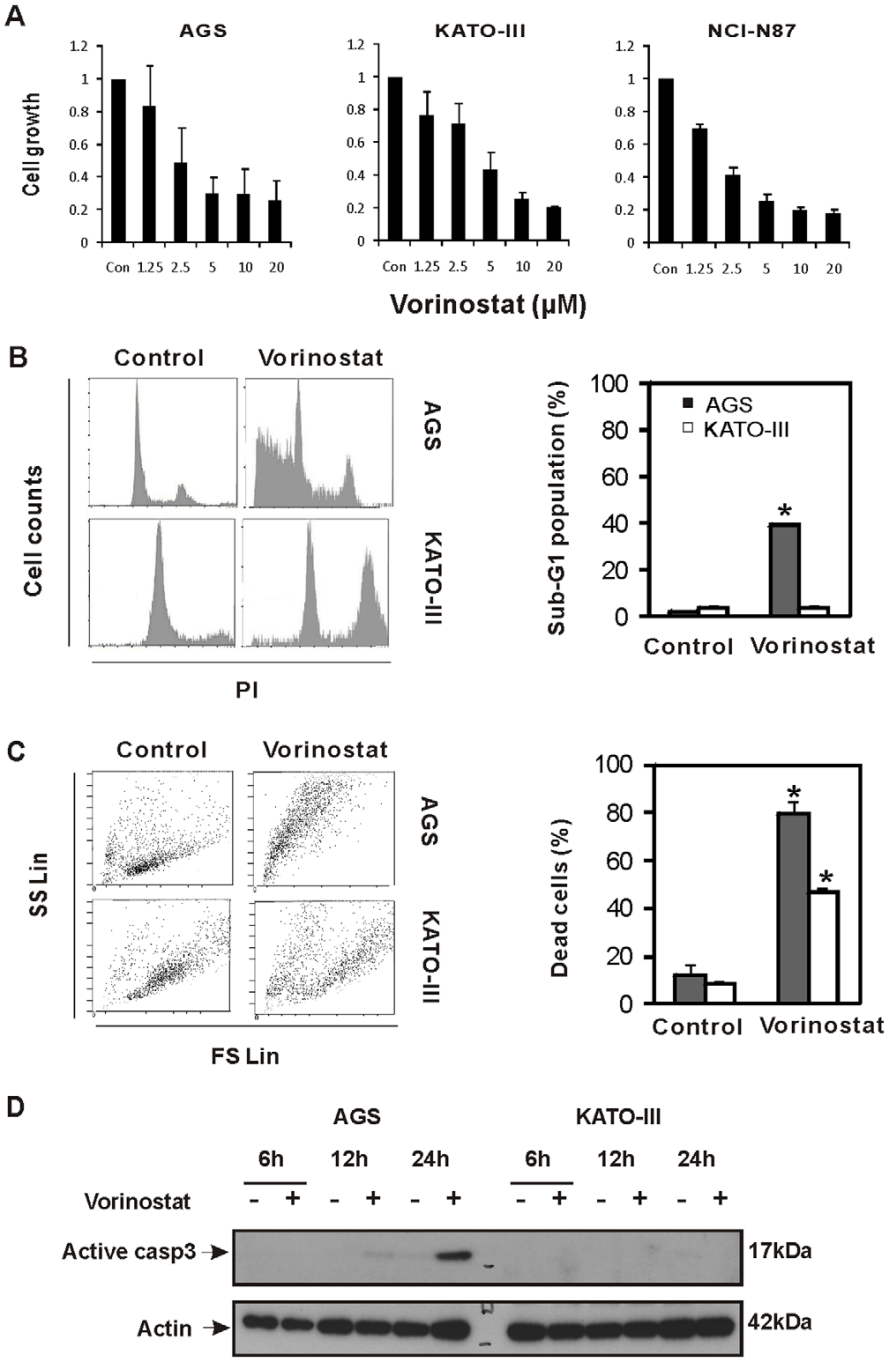
In vitro therapeutic efficacy of vorinostat in gastric cancer cell lines. (A) MTT assays were performed after incubation of AGS, KATO-III, and NCI-N87 with the indicated concentrations of vorinostat for 72 hrs. (B) Change of cell cycle by vorinostat was assessed by fluorescence-activated cell sorting (FACS) analysis of PI stained cells treated with 5 µM vorinostat for 24 hrs. (C) Viability test using PI exclusive solution. AGS and KATO-III cells were treated with 5 µM vorinostat for 72 hrs and assessed by FACS. In representative plot, dead cells were manifested as dots with low forward scatter and high side scatter. (D) Western blot analysis of active caspase-3 from AGS and KATO-III gastric cancer cells without or with 5 µM vorinostat treatment. Cell lysates were analyzed at the indicated time points. Actin was used as a loading control. *, *P*<0.05. In the bar graph, data represent mean+SD (standard deviation).

To evaluate the therapeutic efficacy of vorinostat, we assessed the growth of established gastric cancer cell lines (AGS, KATO-III, and NCI-N87) after 72 h vorinostat treatment using MTT assay. Compared with untreated cells, vorinostat significantly inhibited cell viability in a dose-dependent manner in all gastric cancer cell lines (Figure 1A). We confirmed the reduction in cell viability by cell cycle analysis of AGS and KATO-III cancer cells. We showed that treatment with vorinostat (5 µM) for 24 h induced a marked increase in the sub-G1 proportion of AGS cells compared with control (2.3±0.07% vs 39.2±0.99%, respectively; *P*<0.01), indicating the induction of cell death (Figure 1B). In contrast, the sub-G1 proportion of vorinostat treated KATO-III cells was not affected (Figure 1B), whereas the proportion of G2/M cells increased significantly (21.4±1.91% vs. 29.3±0.35%; *P* = 0.044), indicative for cell cycle arrest. We further tested cell viability using PI-exclusion. We treated AGS and KATO-III cells with 5 µM vorinostat for 72 h and assessed them by flow cytometry (Figure 1C). The amount of dead cells, which have low forward scatter and high side scatter, was significantly increased in AGS cells (12.4±4.3% vs. 79.4±5.7%), and also in KATO-III cells, compared to control cells (8.7±0.6% vs. 46.8±2.1%). We finally analyzed the induction of apoptosis after vorinostat treatment in AGS and KATO-III gastric cancer cell lines using immunoblot. Vorinostat increased apoptosis, as assessed by caspase-3 cleavage, in AGS cells and to a lesser extent in KATO-III cells (Figure 1D).

In summary, these data indicate that vorinostat has antiproliferative effect on gastric cancer cell lines by inducing apoptosis in AGS cells and G2/M cell cycle arrest in KATO-III cells.

### Global gene expression analysis of gastric cancer cell lines AGS and KATO-III after vorinostat treatment

**Figure 2:**
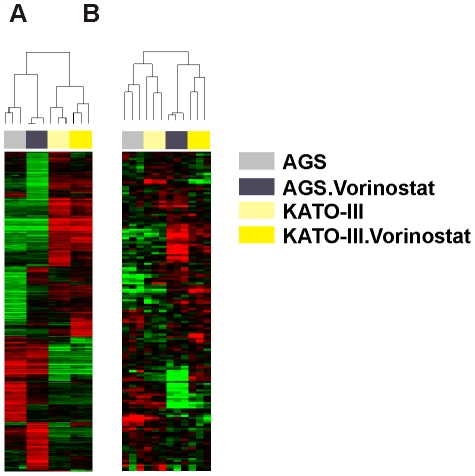
Hierarchical Clustering Gene expression analysis of gastric cancer cell lines after vorinostat treatment. (A) Unsupervised hierarchical clustering of gene expression data from AGS and KATO III before and after 5 µM vorinostat treatment for 48 hours. Genes with an expression level that has at least 2-fold difference relative to median value across cell lines in at least 2 arrays were selected for hierarchical clustering analysis (3,646 gene features). (B) Supervised hierarchical clustering with autophagy related genes (149 probes) of AGS and KATO III after vorinostat treatment.

To analyze the effect of vorinostat on global gene expression, AGS and KATO-III gastric cancer cells were treated with 5 µM vorinostat for 48 hours and microarray analysis was performed. Unsupervised cluster analysis of microarray data after vorinostat treatment showed that AGS and KATO-III cells clustered with the same cell line regardless to vorinostat treatment (Figure 2A). Because autophagy has been reported to have a role in vorinostat-induced effects in other cancers [20], [21], we conducted supervised analysis of the autophagy-related gene set (80 genes and 149 probes; Table S2). Vorinostat-treated gastric cancer cell lines were clustered together (Figure 2B), indicating that induction of autophagy is an important property of vorinostat in gastric cancer lines.

### Inhibition of autophagy enhances vorinostat efficacy in gastric cancer cells

**Figure 3:**
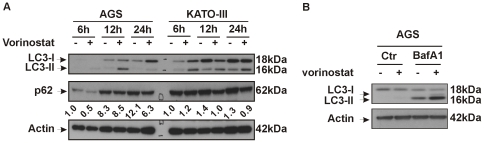
Autophagy induction by vorinostat treatment in gastric cancer cells. (A) AGS and KATO-III were treated with 5 µM vorinostat for the indicated time points. Protein levels of LC3 and p62 were analyzed by immunoblot analysis. Actin was used as a loading control. p62 levels were measured by densitometric analysis of the western blots and compared to actin levels. p62 levels of untreated AGS and KATOIII cells were considered as 1. (B) AGS cells were treated for 12 h with 5 µM vorinostat with or without 50 nM bafilomycin A1 (BafA1). Cell lysates were analyzed by immunoblot analysis for LC3 and actin.

**Figure 4:**
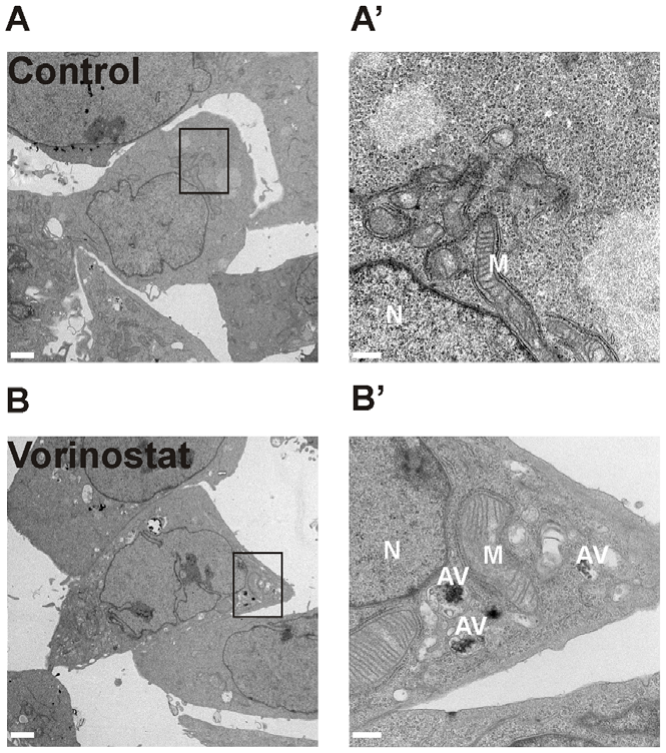
Electron microscopy analysis of autophagy in AGS cells. After 12 h treatment DMSO (A, A') or with 5 µM vorinostat (B, B') (left panels, low magnification, scale bar: 2 µm), EM analysis was performed. High magnification images of boxed areas with Av depicting autophagic vacuoles (left panels, scale bar: 500 nm; N: nucleus, M: mitochondria).

Given that autophagy may have a role in both cancer cell survival and cancer cell death after drug treatment [22], [23], we further evaluated the contribution of this process to the effect of vorinostat on gastric cancer cell lines. We functionally evaluated this process by immunoblot analysis of the autophagy marker microtubule-associated protein 1 light chain 3 (LC3). Vorinostat-treated KATO-III cells, and to a lesser extent AGS cells, showed a clear accumulation of the faster-migrating lipidated form of LC3 (LC3-II) (Figure 3A). Accumulation of LC3-II may result from either upregulation of autophagosome formation or blockage of autophagic degradation of LC3-II [24], [25]. We therefore analyzed vorinostat-treated cells for p62 degradation, a marker of autophagic flux [24], and we observed a decrease in p62 protein levels in vorinostat treated cells compared to the time matched untreated cancer cells (Figure 3A). In addition, cotreatment of vorinostat with bafilomycin A1 (BafA1), an inhibitor of autophagosome-lysosome fusion, further increased LC3-II accumulation (Figure 3B), compatible with vorinostat inducing autophagic flux rather than blocking the degradative capacity of autophagolysomes. Electron microscopy (EM) is a sensitive, quantitative and definitive method for detection of autophagy [24]. Consistent with the western blot data, EM analysis showed that vorinostat markedly increased autophagosome formation in AGS cells (Figure 4B and B') compared to control cells (Figure 4A and A').

**Figure 5:**
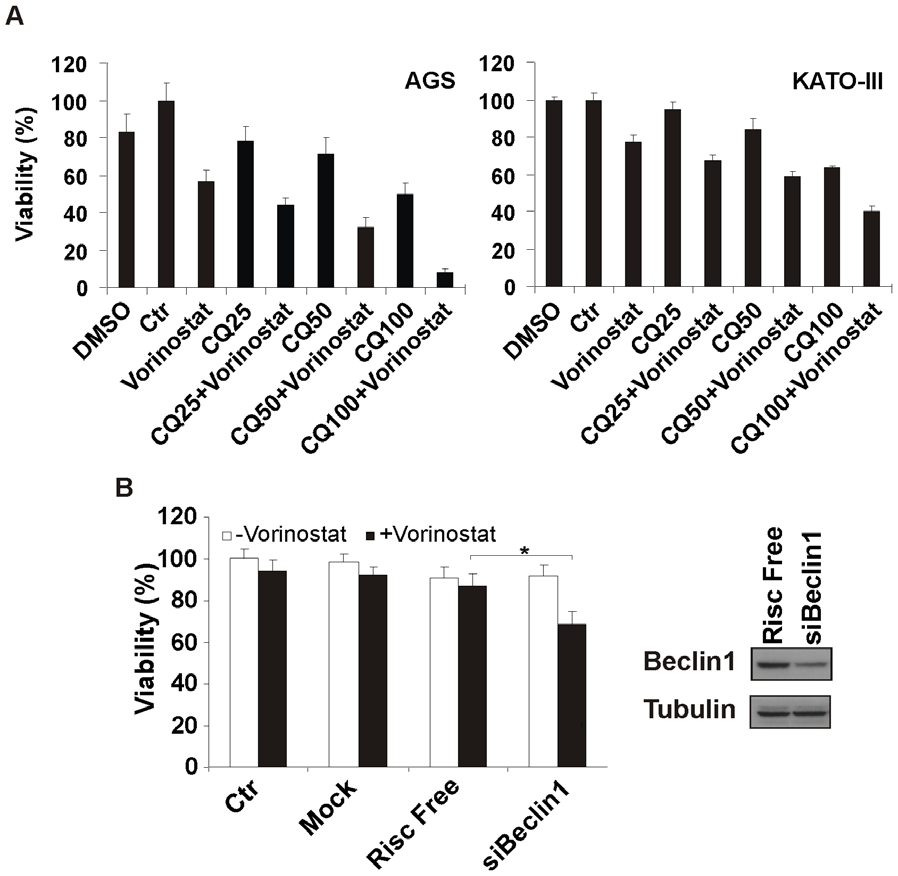
Effect of vorinostat-induced autophagy inhibition. (A) AGS and KATO-III cells were treated with 5 µM vorinostat and different concentrations of chloroquine (CQ). Cell viability was assessed after 12 hours using crystal violet staining and control (Ctr) was set as 100%. (B) KATO-III cells were transfected with siRNA against Beclin 1 (siBeclin1) or with non-targeting Risc Free siRNA or treated with Dharmafect I alone (mock). siRNA efficiency was confirmed by western blot analysis of Beclin 1 and Risc Free as a control. α-tubulin was used to show equal loading of proteins. Viability was measured after 12 hours vorinostat treatment using crystal violet staining. Viability of untreated control (Ctr) cells was set as 100%. *, *P*<0.05.

To investigate whether the accumulation of autophagosomes protects cells against the cellular stress elicited by vorinostat, we inhibited the autophagy process using the pharmacological inhibitor chloroquine and small interfering RNA (siRNA) against beclin-1. Addition of chloroquine to vorinostat-treated AGS and KATO-III cells resulted in a dose-dependent decrease in viability (Figure 5A). The reduction in viability was confirmed in KATO-III cells treated with beclin-1 siRNA (Figure 5B). Together, the data suggest that inhibition of vorinostat-induced autophagy may improve the efficacy of vorinostat treatment of gastric cancer cells.

### Vorinostat changes gene signature in human gastric cancer cells

To understand the effects of vorinostat on gene expression in gastric cancer cell lines, we conducted microarray analysis of vorinostat treated AGS and KATO-III gastric cancer cell lines (Fig. 2). Our analysis revealed significant genomic differences between untreated and vorinostat-treated gastric cancer cells (AGS and KATO-III). After vorinostat treatment, the expression of 1014 genes was increased and the expression of 760 genes was decreased in the AGS cell line. In KATO-III cell line, 164 genes were up and 191 genes were down regulated (two-fold difference; *P*<0.001). Vorinostat altered significantly the expression of 140 genes in both AGS and KATO-III cell lines (vorinostat specific gene signature). The genes which were most altered after vorinostat treatment were identified as *SPANXA1, SPANXA2, VGF, DHRS2, ENTPD8, PNPLA7, STX1A, ARRDC4, KRT13, PRPH, NEU1, TXNIP, CCK* (>4-fold up-regulation) and *MUC1, IFITM1, ANKRD37* (>4-fold down-regulation).

**Figure 6:**
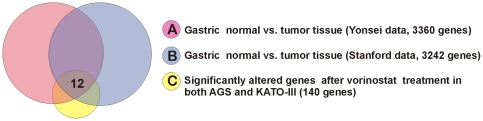
Change of gene expression signature after vorinostat treatment. Venn Diagram of genes selected by univariate test (two-sample t-test). Genes were selected for p<0.001 between compared groups. The red circle (gene list A) represents gastric cancer specific genes from Yonsei data. The blue circle (gene list B) represents gastric cancer specific genes from Stanford data. The yellow circle (gene list C) represents vorinostat specific gene signature from both AGS and KATO-III cell lines (*P*<0.001, 2 fold change).

Next, we intended to identify biomarker candidates which can predict vorinostat sensitivity of human gastric cancer patients. Therefore, we combined the set of altered genes in vorinostat treated gastric cancer cell lines (vorinostat specific gene signature) and the two human gastric cancer signatures generated from the Yosei and Stanford data using Venn diagram analysis. We found that the relative expression levels of 12 genes were reversed by vorinostat treatment (Figure 6). Of these 12 genes, 7 genes highly expressed in gastric cancer tissues were down-regulated (*ITGB5*, *TYMS*, *MYB*, *APOC1*, *CBX5*, *PLA2G2A*, *KIF20A*) and 5 low-expressed genes were up-regulated after vorinostat treatment (*SCGB2A1*, *TCN1*, *CFD*, *APLP1*, *NQO1* (Table 3).

**Table 3:** Reversed genes* after vorinostat treatment in AGS and KATO-III among gastric cancer specific genes**.

Gene symbol	Description
**5 Up regulation after vorinostat treatment**	
SCGB2A1	secretoglobin, family 2A, member 1
TCN1	transcobalamin I (vitamin B12 binding protein, R binder family)
CFD	complement factor D
APLP1	amyloid beta (A4) precursor-like protein 1
NQO1	NAD(P)H dehydrogenase, quinone 1
**7 Down regulation after vorinostat treatment**	
ITGB5	integrin, beta 5
TYMS	thymidylate synthetase
MYB	v-myb myeloblastosis viral oncogene homolog
APOC1	apolipoprotein C-I
CBX5	chromobox homolog 5 (HP1 alpha homolog, Drosophila)
PLA2G2A	phospholipase A2, group IIA
KIF20A	kinesin family member 20A

*More than 2 fold changed genes were selected.

**Gastric cancer specific genes were extracted from GSE13861 data (3360 genes) and GSE13911 data (3242 genes).

## Discussion

Our results indicate that a global human gastric cancer gene signature might be useful to find therapeutic agents that rather target the genomic signature of gastric cancer instead of targeting one or two specific genes. Using the Connectivity Map, we found that HDAC inhibitors, such as vorinostat and trichostatin A, had an inversely correlated gene signature compared to the gastric cancer specific gene signature and therefore may be lead therapeutic candidates for gastric cancer. *In vitro* evaluation of the therapeutic efficacy of vorinostat revealed that this therapeutic drug suppressed growth of different gastric cancer cell lines. Next to its antiproliferative effects, vorinostat also upregulated autophagy-specific genes. Inhibition of vorinostat-induced autophagy resulted in a further reduction of viability. Moreover, combined analysis of gastric cancer cell lines treated with vorinostat and samples of gastric cancer patients showed that vorinostat altered the expression levels of a set of twelve gastric cancer specific genes.

Our findings showed that HDAC inhibitors, vorinostat and trichostatin A, were the top therapeutic candidates for gastric cancer, which agrees with the concept that HDAC is overexpressed in gastric cancer tissues [26]. HDAC inhibitors have been shown to increase acetylation of histones, therefore affecting gene expression. These inhibitors manifest anticancer effects by inducing the intrinsic and extrinsic apoptosis pathway [27], [28], blocking tumor angiogenesis [29], and inhibiting intracellular stress response pathways [30]. Because HDAC inhibitors have global effects on gene expression, they may affect as yet unrevealed cellular processes [31], [32]. In clinical settings, HDAC inhibitors have been mostly applied to hematologic malignancies, but clinical trials in solid tumors are ongoing. A recent study, supporting our *in vitro* findings showed therapeutic efficacy of HDAC inhibitors on human gastric cancer samples using the histoculture drug response assay [33]. However, it remains to be unrevealed whether specific molecular-defined subgroups can predict response or resistance to HDAC inhibitors. In addition, the application of potentially useful biomarkers still has several limitations [34].

The Connectivity Map remains difficult to evaluate until we can assess the extent to which genomic signatures obtained from *in vitro* experiments recapitulate the complexities of human disease. Although the Connectivity Map contains more than 7000 expression profiles representing 1,309 compounds, its approach still deals with some limitations such as the limited amount of cell line data (MCF7, PC3, etc) and the ignorance of microenvironmental influences from the human body [16]. In addition, gene expression profiles derived from the treatment of cultured human cells might not be correlated with *in vivo* anticancer effect, due to the complex nature of cancer. Moreover, the input of only a limited amount of genes is allowed, which may bias the obtained results. Future studies need to be addressed whether our results obtained through cmap analysis can be confirmed in an *in vivo* gastric cancer model. Despite these limitations, Connectivity Map analysis is a potentially useful method for new functions for drugs, including vorinostat, already in use in clinic for other purposes.

*In vitro* evaluation of therapeutic efficacy of vorinostat on gastric cancer cell lines revealed that this therapeutic drug showed an antiproliferative effect at physiological relevant doses (5 µM) compatible with other publications: IC_50_ for AGS and KATO-III has been shown to be respectively 2.9 µM and 5.9 µM [35]. Analysis of cell cycle (Figure 1B), cell viability and (Figure 1C) apoptotic responses (Figure 1D) indicated that the effects of vorinostat show a discrepancy in different gastric cancer cell lines, inducing apoptosis in AGS cells and G2/M arrest in KATO-III cells, respectively. The difference in genetic/mutational background of the cell lines may account for the discrepancy.

Microarray gene expression analysis comparing vorinostat-treated and untreated gastric cancer cell lines indicated that this drug induced autophagy, which is in agreement with previous reports in other cancer cell lines [20], [21]. As both induction and inhibition of autophagy may have therapeutic benefits [22], [23], we further evaluated the role of autophagy in gastric cancer cells after vorinostat treatment. Inhibiting autophagy using a well-known antimalarial drug, chloroquine [21] and using siRNA against beclin-1 resulted in a reduction in viability of gastric cancer cell lines in the presence of vorinostat, suggesting autophagy is activated as a protective survival response after vorinostat treatment (Figure 5). Thus combining anticancer agents such as vorinostat and inhibitors of autophagy could provide a therapeutic advantage in the fight against gastric cancer.

To identify a vorinostat induced gene signature, we evaluated the gene expression profiles of AGS and KATO-III cell lines after vorinostat treatment. The expression of 1774 and 355 genes was reversed (>2-fold, p<0.001) in AGS and KATO-III cell lines, respectively. This suggests that the AGS cell line was more vulnerable than KATO-III to vorinostat treatment in regard to gene expression. This manifestation may explain the sensitivity of AGS cells to vorinostat. We discovered that vorinostat significantly changed the expression of a set of 140 genes in both AGS and KATO-III cell lines. Our results confirm earlier studies showing that that expression of MUC1 and TXNIP is reversed after HDAC inhibitor treatment [36]–[38]. High MUC1 expression is related to poor prognosis and carcinogenesis in gastric cancer [39]. Additionally, re-expression of TXNIP leads to cell growth arrest and apoptosis in cancer [37].

To determine a vorinostat induced gene signature for human gastric cancer, we combined the vorinostat induced cell line gene signature with the human gastric cancer gene signature We found that vorinostat altered expression levels (>2 fold) of only twelve human gastric cancer specific genes, which can potentially explain the mechanism of the vorinostat effect in gastric cancer patients. Expression of *ITGB5, TYMS, MYB, APOC1, CBX5, PLA2G2A,* and *KIF20A* which is up-regulated in human gastric cancer tissues, was significantly decreased by vorinostat. Some of these genes are already known to play a role in carcinogenesis or metastasis of gastric cancer or other cancers. First, expression levels of *TYMS* regulating DNA synthesis and repair showed negative relation to drug sensitivity in gastric cancer and is a marker for drug resistance [40]. Second, *MYB* is known as a proto-oncogene for leukemia, colon cancer, breast cancer, and esophageal cancer [41]–[44], and our results show for the first time that the expression of this gene is also increased in gastric cancer. Third, *APOC1* commonly up regulated in gastric cancer compared to normal gastric epithelium [45], [46], is already considered as a possible biomarker for gastric cancer. Last, *KIF20A* plays a role in cytokinesis and inhibiting expression of this gene attenuated growth of pancreatic cancer cells [47]. In contrast, vorinostat treatment also increased the expression of genes which are normally low in human gastric cancer tissue, such as *SCGB2A1, TCN1, CFD, APLP1*, and *NQO1*. *CFD*, known to be important in immune defense in tissue [48], possibly participates in inflammation of gastric epithelium [49]. *APLP1*, a transcriptional target of p53, may be involved in cell death [50] and therefore vorinostat-induced expression of *APLP1* may contribute to apoptotic cell death observed in our study. *NQO1* inactivation has been associated with increased susceptibility to a variety of carcinogens [51], and has been related to the risk of colorectal cancer [52], [53]. Further experiments are required to understand the functions of these genes and their relation to the effects of vorinostat (i.e. induction of autophagy and cell death) in gastric cancer. However, they are feasible to be considered as predictive biomarkers for vorinostat sensitivity. One of the clinical implications of this ‘12 gene signature’ is that it can be used to select gastric cancer patients who may benefit from this treatment.

In summary, we generated a global human gastric cancer gene signature and by using Connectivity Map we found that the HDAC inhibitor vorinostat is a strong candidate therapeutic agent for gastric cancer. We presented that gene signature analysis may be useful in discovering therapeutic agents for the treatment of gastric cancer, such as vorinostat, which may be a promising therapeutic agent alone or in combination with autophagy inhibitors.

## Supporting Information

Figure S1 **Gene expression signature (3,360 genes) of human gastric cancer.** Measured gene expression values were log 2-transformed and median-centered across samples before generating the heatmap. The data are presented in matrix format in which rows represent individual gene and columns represent each tissue. The red and green color in cells reflects relative high and low expression levels respectively.
(TIF)

Table S1 **A list of 500 up regulated and 500 down regulated genes in gastric cancer, used as input query into Connectivity Map.**
(XLS)

Table S2 **A list of 80 autophagy-related genes (149 probes), used for supervised analysis (** **Figure 2B** **).**
(XLS)
