# Supplementary figures and images for: Gene Expression Signature Analysis Identifies Vorinostat as a Candidate Therapy for Gastric Cancer
Source: PLoS One. 2011 Sep 9;6(9):e24662. doi: 10.1371/journal.pone.0024662 (PMC3170379; doi:10.1371/journal.pone.0024662)

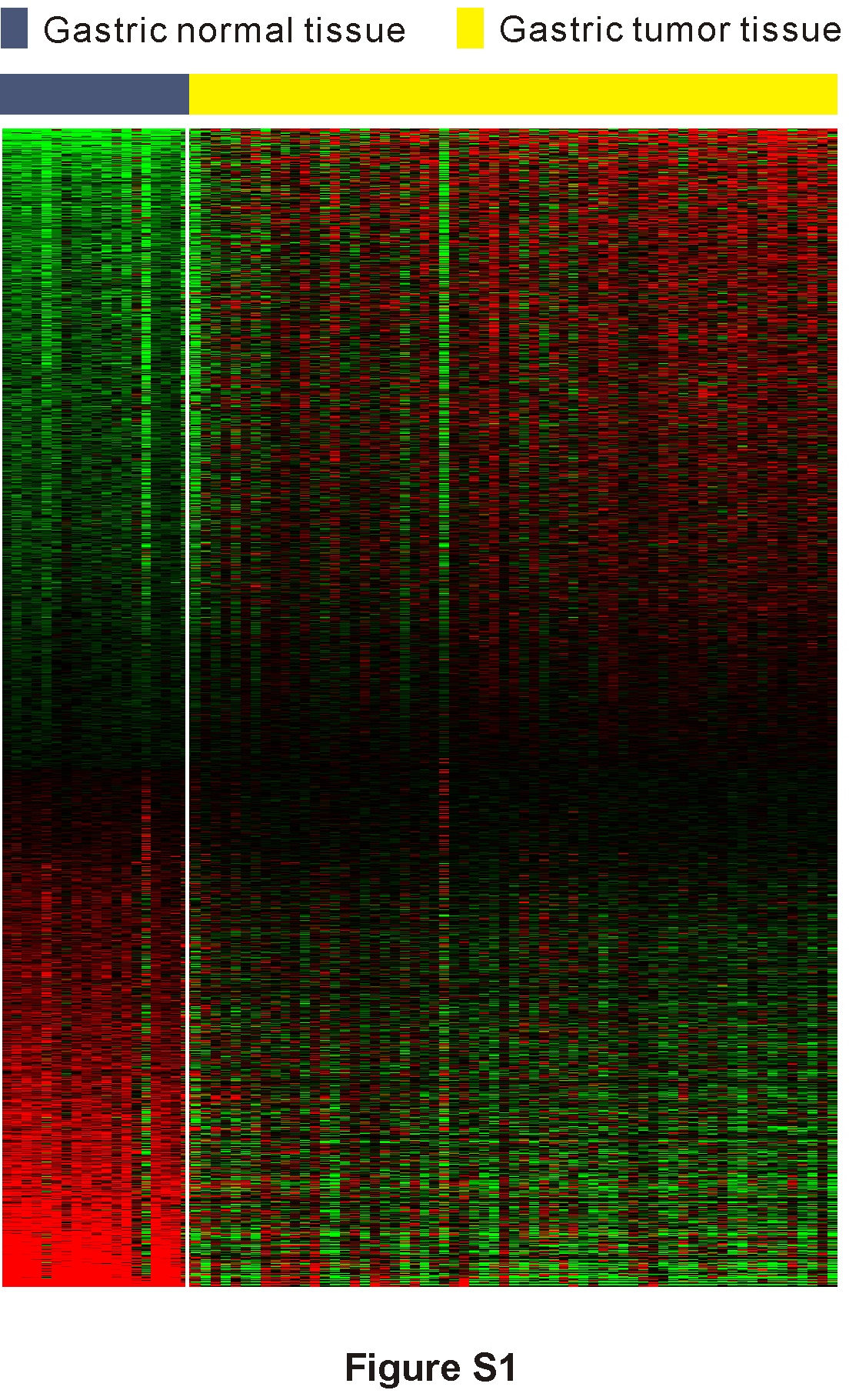

Supplement: Figure S1 — Gene expression signature (3,360 genes) of human gastric cancer. Measured gene expression values were log 2-transformed and median-centered across samples before generating the heatmap. The data are presented in matrix format in which rows represent individual gene and columns represent each tissue. The red and green color in cells reflects relative high and low expression levels respectively. (TIF) [file pone.0024662.s001.tif]
